# Identification of novel major and minor QTLs associated with *Xanthomonas oryzae* pv. *oryzae* (African strains) resistance in rice (*Oryza sativa* L.)

**DOI:** 10.1186/s12284-016-0090-9

**Published:** 2016-04-23

**Authors:** Gustave Djedatin, Marie-Noelle Ndjiondjop, Ambaliou Sanni, Mathias Lorieux, Valérie Verdier, Alain Ghesquiere

**Affiliations:** Université Polytechnique d’Abomey, BP 2282 Abomey, Benin; Africa Rice Center (AfricaRice), 01 BP2031 Cotonou, Benin; IPME Interactions Plantes Microorganismes, Environnement, IRD - Cirad - University Montpellier, 34394 Montpellier, France; UMR Diversité, Adaptation et Développement des plantes (DIADE), Institut de Recherche pour le Développement, 911 Avenue Agropolis BP 64501, 34394 Montpellier Cedex 5, France; Université d’Abomey Calavi, 01 BP526 Cotonou, Benin

**Keywords:** Molecular mapping, QTL, disease resistance, *Xanthomonas oryzae* pv. *oryzae*, *Oryza sativa*

## Abstract

**Background:**

*Xanthomonas oryzae* pv. *oryzae* (*Xoo*) is the causal agent of Bacterial Leaf Blight (BB), an emerging disease in rice in West-Africa which can induce up to 50 % of yield losses. So far, no specific resistance gene or QTL to African *Xoo* were mapped. The objectives of this study were to identify and map novels and specific resistance QTLs to African *Xoo* strains.

**Results:**

The reference recombinant inbred lines (RIL) mapping population derived from the cross between IR64 and Azucena was used to investigate *Xoo* resistance. Resistance to African and Philippine *Xoo* strains representing different races was assessed on the RIL population under greenhouse conditions. Five major quantitative trait loci (QTL) for resistance against African *Xoo* were located on different chromosomes. Loci on chromosomes 1, 7, 9, 10 and 11 explained as much as 13 %, 37 %, 13 %, 11 % and 15 % of resistance variation, respectively. A major novel QTL located on chromosome 7 explained 37 % of the phenotypic variance to the African *Xoo* corresponding to race A3 whereas that on chromosome 11 is effective to all African races tested. Together with genes and QTLs for resistance to bacterial blight previously described, the QTLs described here were mapped onto the reference *O. sativa* subs japonica (var. Nipponbare) physical map.

**Conclusion:**

We characterized new resistance QTLs. While some co-localize with known resistance genes/QTLs to Asian strains, others are specific to African strains. We result with new information on genes and QTLs for resistance to bacterial blight that will be useful for controlling the disease.

## Background

Rice is a staple food for much of the world’s population, including that of sub-Saharan Africa. Population growth and migration have recently lead to a rapid growth in rice consumption in dozens of African countries. With the recent expansion and intensification of rice cultivation in Africa, rice diseases have concomitantly increased in most African rice growing areas (Sere et al. 2005; Traoré et al. 2009). Bacterial Blight (BB), caused *by Xanthomonas oryzae* pv*. oryzae* (hereafter, *Xoo*), is one of the most serious rice bacterial disease in Africa. The disease is prevalent in irrigated and rain-fed lowland rice growing areas. BB was first reported in Mali in 1979 and later in Senegal, Niger, Nigeria, Gabon, Mauritania, Benin, Burkina and Cameroon. Over the last decade a significant recurrence of this disease was observed in several regions in Africa (Reckhaus et al. 1983; Gonzalez et al. 2007; Basso et al. 2011; Verdier et al. 2011).

Resistance *(R)* genes are largely used in rice breeding programs in Asia to control BB disease. More than 30 *R* genes, which are given the prefix Xa for Xanthomonas, have been identified so far (for a review see Verdier et al. 2011) among which a few are deployed in breeding programs. Important prerequisites to the deployment of *R* genes are as follows: 1) to have an intensive knowledge of *Xoo* population structure, race distribution and frequency 2) to determine the durability of resistance of *R* genes to be deployed.

Most of the BB *R* genes provide complete race-specific resistance to *Xoo* strains. Different combinations of *Xa4, xa5, Xa7, xa13* and *Xa21* have been incorporated in popular rice commercial varieties in different countries in Asia (Century et al. 1999; Singh et al. 2006; Swamy et al. 2006; Perez et al. 2008; Sundaram et al. 2009, Shanti 2010; Suh et al. 2013; Ruengphayak et al. 2015). Few examples indicate that some R genes used for controlling BB disease are overcome by virulent strains as shown in Korea with the resistant gene *Xa21* (Lee et al. 1999; Zhang et al. 2006). *Xa4* is a gene used for more than 30 years and has introgressed in high yielding varieties in Asia but has lost efficacy in many cultivated areas (Mew et al. 1992). Although durability of BB *R* genes is, in part, because mutation of *Xoo* to overcome R genes (Vera Cruz et al. 2000), recent field and laboratory studies have also shown the influence of temperature on the interactions of rice *R* gene with *Xoo*. High temperatures are conducive to BB disease, and most BB *R* genes, including *Xa4*, are less effective at controlling BB disease at high temperatures (Vera Cruz et al. 2000; Webb et al. 2010).

*Xanthomonas oryzae* (*Xo*) is a diverse species, with distinct phylogenetic lineages comprising US *Xo*, Asian *Xoo*, African *Xoo*, and *Xanthomonas oryzae* pv. *oryzicola* (*Xoc*) (Triplett et al*.*^2011^; Hajri et al. 2012). Another lineage improperly named *Xanthomonas campestris* pv. *leersia*e (Xcl) comprises strains isolated on weeds (Wonni et al. 2014). Previous work highlighted differences in the race structure between Asian and African *Xoo* strains (Gonzalez et al. 2007). Virulence assays revealed three races (A1, A2 and A3) present in Mali, Burkina-Faso, Niger and Cameroon that do not represent any of the known *Xoo* races characterized in Asia so far (Gonzalez et al. 2007; Triplett et al. 2011). According to experiments conducted on BB isogenic lines (IRBB), BB resistance genes *Xa4, xa5* and *Xa7* provide resistance to some African *Xoo* strains (Gonzalez et al. 2007). Although in absence of a complete overview of *Xoo* race prevalence in Africa, we anticipated that *Xa4, xa5* and *Xa7* could provide resistance against strains of *Xoo* in Burkina-Faso, Cameroun and Niger. Despite the increasing importance of BB in Africa, little is known on the genetic determinism of resistance. *O. glaberrima* and *O. sativa* accessions were screened for resistance to African *Xoo* strains. The tropical japonica landrace Azucena is susceptible to all African *Xoo* strains. Few accessions, among them the indica cultivar IR64, are highly resistant to African *Xoo* strains. None of these accessions had the *xa5* or *Xa21* resistance alleles (Djedatin et al. 2011) suggesting that these accessions carry new resistance genes that could be good targets for R gene discovery and further deployment.

With the completion of genome sequences for *japonica* and *indica* rice (Kawahara et al. 2013) and for *O. glaberrima* (Wang et al. 2014a, b), it is essential to have a better picture of the different *Xa* resistance genes and QTLs characterized so far and their positions in the rice genome.

The objectives of this study are to:

1. Identify and analyse the genetic basis of rice resistance to African *Xanthomonas oryzae* pv. *oryzae* strains by developing a QTL approach using the reference mapping population made of recombinant inbred lines (RIL) derived from the cross between IR64 and Azucena.
2. Map novel and known bacterial blight resistance genes and QTLs to *Xoo* strains and analyze their colocalization on the reference Nipponbare physical map.

For the first time in history, we report on specific resistance QTLs to African *Xoo* strains. These QTLs will be used in breeding program to enhance rice genetic resistance to BB in Africa.

## Results

### Study of inheritance of BB resistance

The average lesion length induced by *Xoo* African strains on IR64 and Azucena are respectively: 0.25 ± 0.1 and 16.1 ± 2 cm with *Xoo* MAI1; 0.76 ± 0.2 and 22.36 ± 2.7 cm with *Xoo* BAI4; 4.07 ± 1 and 22.29 ± 3 cm with *Xoo* BAI3; 2.07 ± 0.5 and 26.29 ± 3.3 cm with *Xoo* NAI8. IR64 is observed to be highly resistant and Azucena highly susceptible to all virulent strains of African *Xoo* tested so far. The disease scores of the RILs range widely, from as low as 0.12 cm to as high as 30.05 cm, 0.14 to 32.2 cm, 0.2 to 32.6 cm and 0.44 to 37.9 cm with *Xoo* strains MAI1, BAI4, BAI3 and NAI8, respectively (Table 1). This continuous variation of lesion lengths indicates the existence of QTLs underlying the segregation of resistance. Both parents, IR64 and Azucena, are susceptible to Asian *Xoo* strain PXO86 with an average lesion length of 16.46 ± 1.5 and 26 ± 3 cm, respectively. Conversely, IR64 is resistant to PXO61; the Philippines race 1, with an average lesion length of 1.92 ± 0.4 cm, whereas Azucena is susceptible with an average lesion length of 28.32 ± 3.1 cm. The lesion length of the 178 RILs lines shows a continuous variation with an average lesion length of 6.95 to 34.5 cm and 0.5 to 30.8 cm with PXO86 and PXO6, respectively (Table 1), indicating the resistance to Asian strains is controlled by QTLs.

**Table 1:** Lesion length induced by African and Asian *Xoo* on recombinant inbred lines and their parents

*Xoo* strains	Lesion length (cm) induced on		
	IR64	Azucena	RIL
MAI1	O.25 ± 0.1	16.1 ± 2	0.12 to 30.05
BAI4	0.76 ± 0.2	22.36 ± 2.7	0.14 to 32.2
BAI3	4.07 ± 1	22.29 ± 3	0.2 to 32.6
NAI8	2.07 ± 0.5	26.29 ± 3.3	0.44 to 37.9
PXO86	16.46 ± 1.5	26.0 ± 3	6.95 to 34.5
PXO61	1.92 ± 0.4	28.32 ± 3.1	0.5 to 30.8

*Xoo Xanthomonas oryzae* pv. *oryzae*, *RIL* Recombinant Inbred Lines

### Mapping QTLs using SSR markers

The IR64 x Azucena genetic map used in this study were comprised of 226 SSR markers and covered 1652.06 cM of the genome with an average inter-marker interval of 7.31 cM. QTL mapping, based on ANOVA, evidences twelve putative QTLs induced by African *Xoo* strains (MAI1, BAI4, BAI3 and NAI8). Two of them (*qABB-7* and *qABB-11*) have a large effect on chromosomes 7 and 11, respectively. The others induce small effect on chromosomes 1, 3, 4, 8, 9, 10. The composite interval mapping using WinQTLCartographer 2.5 reveals five specific QTLs underlying resistance to African *Xoo* strains including those detected by ANOVA on chromosomes 1, 7, 9, 10 and 11. The estimated additive effect indicates that these loci derive from the resistant parent IR64. *qABB-1*, specific to MAI1, is linked to SSR markers RM129 and RM493, with a LOD score of 4.72 and a percentage of variance explained (R^2^) of 8 %. *qABB-7* is linked to RM125 and RM214 with a LOD score of 16.20 and R^2^ of 30 % for MAI1, and a LOD score of 13.98 and R^2^ of 30 % for BAI4. *qABB-11* is close to RM224 and RM144 with a LOD score of 5.32, 5.87 and 4.18 with *Xoo* MAI1, BAI3 and BAI4, respectively. *qABB-11* controls 7, 9, and 14 % of the phenotypic variation explained by *Xoo* BAI4, MAI1, and BAI3, respectively. The Inclusive Composite Interval Mapping using Qgene-4.3.0, the more accurate QTL analysis method, confirms five specific QTLs mapped on chromosomes 1, 7, 9, 10 and 11 underlying resistance to African *Xoo* strains. The estimated additive effects confirm that these loci derive from the resistant parent IR64. Additive effect, linked markers, LOD score value and PVE are summarized in Table 2. *qABB-11* on chromosome 11 was involved in the resistance to all African *Xoo* strains tested so far. QTLs on chromosomes 9 (*qABB-9*) and 10 (*qABB-10*) are specific to *Xoo* strain NAI8 (race A1). Asian *Xoo* strains induce five resistance QTLs different from those induced by African’s strains except that on chromosome 11. This is also induced by *Xoo* strain PXO61 (Philippines race 1). Three resistance QTLs to *Xoo* strain PXO86 (Phil race 2) are mapped on chromosomes 5 (*qBB-5*), 8 (*qBB-8*) and 12 (*qBB-12*) (Table 2). As indicated by the estimated additive effects, the QTLs on chromosomes 5 and 8 are controlled by Azucena allele while that on chromosome 12 is underlined by IR64 allele. A resistance QTL (*qBB-4*) is detected on chromosome 4 for the Asian *Xoo* strain PXO61.

**Table 2:** Novel QTLs mapped in IR64 x Azucena population using African and Asian *Xanthomonas oryzae pv.oryzae*

*Xoo* ^*a*^ strains	Country of origin	QTL localization: chromosome	QTL name	LOD^*b*^ score	Closely Linked marker	Marker position (cM)	Additive effect	Donor Allele	PVE^c^ (%)
MAI1	Mali	1	*qABB-1*	5.068	RM129	58.45	1.7	IR64	13.4
		7	*qABB-7*	16.006	RM125	5.75	3.2	IR64	36.6
		11	*qABB-11*	4.666	RM144	100.21	1.5	IR64	12.4
BAI4	Burkina Faso	7	*qABB-7*	13.943	RM125	5.75	3.5	IR64	33.4
		11	*qABB-11*	4.68	RM144	100.21	1.8	IR64	12.8
BAI3	Burkina Faso	11	*qABB11*	5.728	RM144	100.21	2.5	IR64	15.3
NAI8	Niger	9	*qABB-9*	4.359	RM242	69.28	2.3	IR64	12.9
		10	*qABB-10*	3.606	RM294A	74.36	-2.23	Azucena	10.8
		11	*qABB-11*	6.03	RM144	100.21	2.7	IR64	17.4
PXO86	Philippines	5	*qBB-5*	4.209	RM440	70.61	-1.68	Azucena	11.5
		8	*qBB-8-2*	4.957	RM281	129.96	-2.16	Azucena	13.4
		12	*qBB-12*	4.869	RM512	38.32	1.9	IR64	13.2
PXO61	Philippines	4	*qBB-4*	3.403	RM252	84.70	-1.7	Azucena	10
		11	*qABB-11*	36.78	RM144	100.21	7.3	IR64	67.9

*Xoo Xanthomonas oryzae* pv. *oryzae, LOD* logarithm of odds, *PVE* Percentage of variance explained

### Heredity studies

The screening of the recombinant inbred lines using Asian *Xoo* strain PXO61 evidenced the same QTL previously induced on chromosome 11 (*qABB-11*) by all African *Xoo* strains (with LOD score = 36.78 and R2 = 67) (Table 2).

The segregation ratio obtained by screening F_2_: IR24 x IRBB4 population with the Asian *Xoo* strain PXO61 is 3 resistant for 1susceptible. This is the segregation ratio of a dominant gene in a F_2_ population. It was the *Xa4* dominant gene which was specific to PXO61. On the contrary, the segregation studies in the same population using the African *Xoo* strain BAI3 revealed a ratio of 3 susceptible for 1 resistant, that is the segregation ratio of a recessive gene in a F_2_ population. Then, the African *Xoo* strain BAI3 induces a recessive gene at the locus of *qABB-11*.

**Fig. 1:**
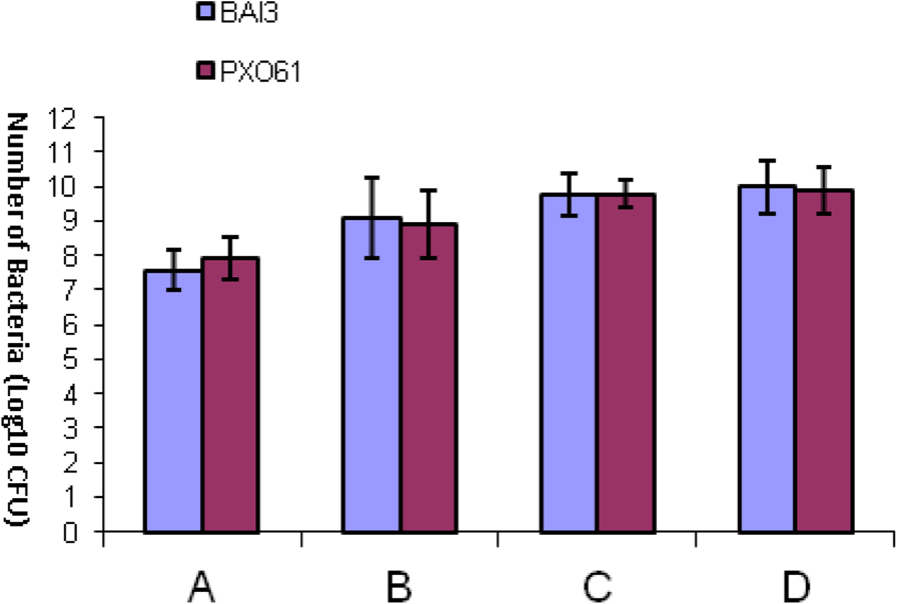
Bacteria growth during twelve days after inoculation of IR24 rice variety. A = 1^st^ fragment of 5 cm (from the inoculation point) of infected leaves. B = 2^nd^ fragment of 5 cm (from 5 to 10 cm to the inoculation point) of infected leaves. C = 3^rd^ fragment of 5 cm (from 10 to 15 cm to the inoculation point) of infected leaves. D = 4^th^ fragment of 5 cm (from 15 to 20 cm to the inoculation point) of infected leaves

**Fig. 2:**
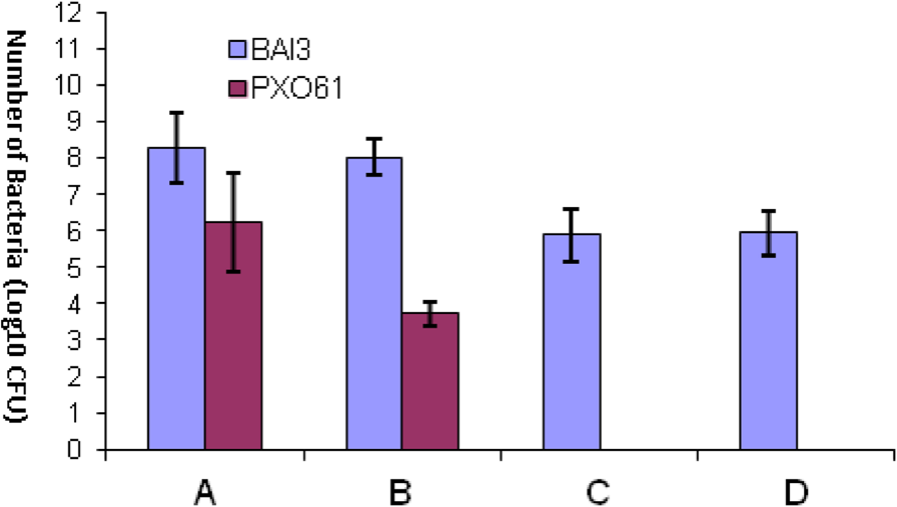
Bacteria growth during twelve days after inoculation of rice isogenic line IRBB4. A = 1^st^ fragment of 5 cm (from the inoculation point) of infected leaves. B = 2^nd^ fragment of 5 cm (from 5 to 10 cm to the inoculation point) of infected leaves. C = 3^rd^ fragment of 5 cm (from 10 to 15 cm to the inoculation point) of infected leaves. D = 4^th^ fragment of 5 cm (from 15 to 20 cm to the inoculation point) of infected leaves

The study of bacterial growth and of the xylem colonization speed by the bacteria shows that both strains PXO61 and BAI3 grown in the same way in IR24 rice variety which is susceptible to bacterial blight. This fact is expressed by a more or less equal number of PXO61 and BAI3 colonies detected at each time. It’s the case on the twelfth day after inoculation when the number of PXO61 and BAI3 colonies is about Log10 (8) in the fraction A and Log10 (9) in the fraction B. This number increases to Log10 (10) and Log10 (10.5), respectively, in the fractions C and D (Fig. 1). On the other hand, in the resistant IRBB4 isogenic line, the colonies number of these two strains is far inferior to those got in IR24 variety (Fig. 2). In this case, both strains grown unequally. On the 12th day after inoculation, we count Log10 (8) of BAI3 and Log10 (6) of PXO61 in the fraction A, Log10 (8) of BAI3 against Log10 (4) of PXO61 in the fraction B. The Asian *Xoo* strain PXO61 is then stopped at the level of fraction B, which is expressed by the absence of colonies in the fractions C and D. On the other hand, Log10 (6) colonies of the African *Xoo* strains BAI3 have been shown in the fraction C and D (Fig. 2).

The results of the quantification and of bacterial kinetic show clearly that the African *Xoo* strain BAI3 is quite different from the one of the Asian *Xoo* strain PXO61 that lights a dominant gene which was the *Xa4* gene. In fact, in the IRBB4 isogenic line, resistant to both strains, the lesion caused by BAI3 was three times bigger than the one caused by PXO61. *Xa4* prevent the multiplication and the progression of PXO61 in the vascular tissues which is expressed on the one hand by the reduced number of PXO61 in these tissues and, on the other hand by the total absence of this bacteria 10 cm to the inoculation point. This is in accordance with the functioning of dominant resistance gene. On the other hand, BAI3 colonies have been observed even at 20 cm to the inoculation point. This strain managed to multiply and to colonize the vascular tissues thus leading to a less resistant phenotype (Fig. 2 ). The African *Xoo* strain BAI3 would thus induced a recessive gene.

## Discussion

In this study, novel resistance QTLs to BB are identified and mapped. Moreover, the information on known bacterial blight genes/QTLs characterized so far was updated (Additional file 1) and their genetic and physical localization positioned on the reference rice physical map (var Nipponbare).

IR64 has been confirmed as highly resistant to African *Xoo* strains. The IR64 x Azucena derived mapping populations segregated for BB resistance. Novel QTLs were discovered, mapped to chromosomes 1, 4, 5, 7, 8, 9, 10, 11, 12. Most were of relatively small effect except on chromosomes 7 and 11. *qBB-4*, *qBB-5*, *qABB-9* and *qBB-12* mapped to genomic regions where BB resistance QTL and or *Xa* genes were previously characterized in other crosses, indicating that BB-resistance genes to Asian and African *Xoo* strains may be shared by several rice accessions. Out of five, four QTLs induced by African *Xoo* strains are different from those induced by the Philippines *Xoo* strain PXO61 indicating these strains induced different resistant genes. QTLs induced by African *Xoo* strains are underlined by the allele of the indica rice IR64 while Azucena (*japonica)* is the donor parent of resistance against Asian *Xoo* strains except with strain PXO61.

The genetic characterization of *Xoo* strains together with the recent advances in *X. oryzae* genomic studies indicate that African *X. oryzae* pv. *oryzae* strains form a separate group within the *X. oryzae* (Gonzalez et al. 2007; Triplett et al. 2011; Hajri et al. 2012; Wonni et al. 2014). Also their repertoire of transcription activator-like (TAL) effectors is reduced compared to the Asian *Xoo* one (Gonzalez et al. 2007).

Two resistance QTLs with main effects were identified. The first, *qABB-7*, induced by the African *Xoo* strains MAI1 and BAI4 on chromosome 7 and the second, *qABB-11*, induced on chromosome 11 by all African strains tested so far are particularly interesting. The first one, *qABB-7*, controls 37 % of the phenotypic variance with a high LOD score of 16 (Table 2) compared to 2.5 to 3 fixed LOD score in other QTL studies (Wang et al. 2006a, b; Sakraborty and Zeng 2011). Our preliminary data indicate that the single resistance genes *Xa4, xa5* and *Xa7* provide strong levels of resistance to African *Xoo* strains collected in the 1980’s and in 2003 (Gonzalez et al. 2007). *Xa4* is located in the *qABB-11* region. The fine mapping of *qABB-7* and *qABB-11* is in progress. Our study also reveals the difference between African and Asian *Xoo* strains in terms of virulence. The resistant and susceptible phenotypes of the parents (IR64 and Azucena) induced by African *Xoo* strains greatly contrast with those induced by Asian *Xoo* strains (Table 1). According to the previous results, resistance to BB depends on the rice genetic background. *O. sativa* subspecies *indica* appeared to be the best source of resistance to bacterial blight in rice conversely to the African cultivated rice *O. glaberrima* that showed a relative narrow resistance basis to BB (Djedatin et al. 2011).

Preliminary segregation and bacteria growth studies revealed that the two most important QTLs induced by African *Xoo* on rice chromosomes 11 are recessive while most of the resistance genes characterized so far are dominant with the exception of *xa5, xa8, xa13, xa19, xa20, xa24, xa33(t), xa34(t)* and *xa35(t)* (Verdier et al. 2011). These results are compatible with the hypothesis that African and Asian strains have different effector genes that induced different resistance genes (Yu et al. 2011a, b; Hajri et al. 2011).

So far, 40 R genes and 17 QTLs conferring host resistance against various strains of *Xoo* have been identified (Li et al. 1999; Chen at al. 2002; Gu et al. 2004; Blair et al. 2003; Ramalingam et al. 2003; Wu et al. 2008a, b; Cheema et al. 2008; Ruan et al. 2008; Korinsak et al. 2009; Wang et al. 2009; Sundaram et al. 2009; Chen et al. 2011; Bhasin et al. 2012; Han et al. 2014; Kim et al. 2015). Approximately one third of naturally occurring R genes against *Xoo* (*xa5, xa8, xa13, xa15, xa19, xa20, xa24, xa26, xa28, xa31(t), xa33(t)* and *xa34*) are recessive (Sanchez et al. 1999; Wu et al. 2008a, b; Ruan et al. 2008; Korinsak et al. 2009). More than 20 R genes were mapped onto rice chromosomes, and some of them have been well characterized. Nine resistance genes have been molecularly cloned including six dominant genes, *Xa21* (Song et al., b)*, Xa1* (Yoshimura et al. 1998), *Xa3/Xa26* (Sun et al. 2004; Xiang et al. 2006), *Xa27* (Gu et al. 2004; Bimolata et al. 2013), *Xa10* (Tian et al*.*^2014^), *Xa23 (*Wang et al. 2014a, b*)* and three recessive *xa5* (Iyer and McCouch 2004) and *xa13* (Chu et al. 2006a, b), *xa25* (Liu et al. 2011) have been cloned. All known resistance genes/QTLs and their flanking markers mapped on the reference Nipponbare physical map show that they are unequally distributed on rice chromosomes. Chromosomes 4 and 11 appeared to carry most of the known BB resistance genes. Indeed, these chromosomes are known to carry clusters of resistance genes analogs (RGA) (Mago et al. 1999; Ghazi et al. 2009). Resistance QTLs to rice sheath blight resistance (Zou et al. 2000) and resistance genes to blast (Wang et al. 1994) also clustered on chromosome 11. Many QTLs mapped closely to single dominant or recessive *Xa* genes. It is the case of *Xa1* and *Xa2* which bracketed *AQBT008* on chromosome 4. Some of them are considered as a single gene like *AQBT023* designated as *Xa4* on the Lemont x Teqing map (Li et al. 1999). After interpolation on the Nipponbare physical map, *AQBT023* was shown to be distinct to *Xa4* located on chromosome 11.

**Fig. 3:**
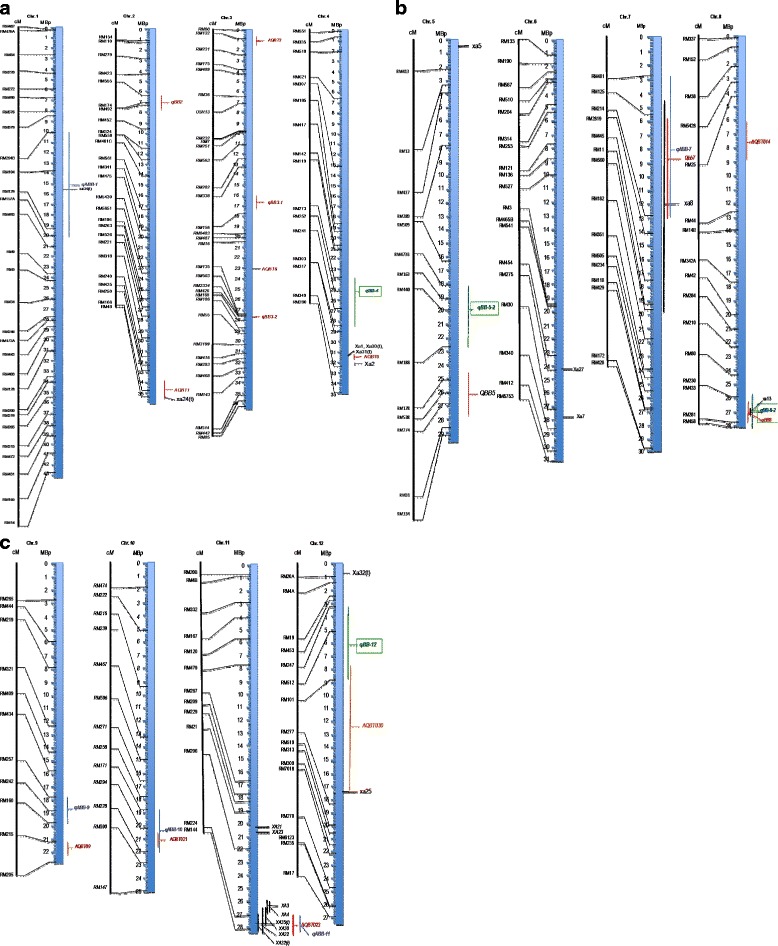
**a**, **b** and **c**: Integrative map showing all known resistance QTLs/genes to African and Asian *Xoo*. The vertical graduated blue thick lines represent the physical map of each chromosome and is linked to the genetic map on the left showing the microsatellites markers (RM). On the right size of each physical map the genes and QTLs are indicated in red, black and green. In black are the R genes that were previously identified with Asian *Xoo* strains. R genes that were cloned are indicated by a horizontal line to a specific location on the physical map. The genes that are not cloned yet are represented by a vertical line indicating the confidence interval. In red we indicated QTLs that were detected in others rice breeding populations. Novel QTLs identified using the African *Xoo* strains are represented in blue those induced by the Philippines strains PXO61 and PXO86 are shown in green

This interpolation on the reference Nipponbare physical map highlights common, specific and novel QTL/genes for resistance to African and Asian *Xoo* strains. Indeed, *xa34(t)* resistance gene to Chinese *Xoo* races V co-localize with qABB-1, the resistance QTL induced by the African *Xoo* strain MAI1 on rice chromosome 1. In the same way, *AQBT021* effective on Asian *Xoo* strains, co-localize with *qABB-10*, a resistance QTL induce by the African *Xoo* strain NAI8 on chromosome 10. *xa8* (Ogawa and Yamamoto 1987), *qBB7* (QTL identified on chromosome 7, Ramalingam et al. 2003) and *qABB-7* overlap on chromosome 7 as well as *xa24* (Wu et al. 2008a, b) and *AQBT001* on chromosome 2. Lemont and Azucena, the donors’ parents of *AQBT021* and *qABB-10* respectively, belong to the japonica subspecies. These resistance QTLs may be the same. On chromosome 8, *xa13*, *qBB8* and *qBB-8-2* co-localize perfectly. *qBB8* and *qBB-8-2* may be underlined by the recessive *xa13* gene, but the heredity of *qBB8* and *qBB-8-2* has not been studied yet. On chromosome 11, the QTL induced by African *Xoo* strains co-localized with *Xa4, Xa3, Xa32* and *xa35(t)*, the known single resistance genes to Asian *Xoo* strains. These *Xa* genes indicated that some major R genes also contribute to quantitative resistance as reported in common bean in which RGAs co-localized with anthracnose-specific QTL (Geffroy et al. 2000). This was also the case of QTL associated with resistance to stripe rust and Barley yellow dwarf virus in barley (Toojinda et al. 2001), and partial resistance to Cucumber mosaic virus in pepper (Pflieger et al. 1999). Specifics and novels QTLs inducing resistance to African and/or Asian *Xoo* strains are identified as *qABB-9* induced by the African *Xoo* strain NAI8 on chromosome 9, *qBB-4* and *qBB-5* induced on chromosomes 4 and 5 by Asian *Xoo* strains PXO61 and PXO86 respectively, (Fig. 3).

In addition to specific resistance QTLs to African *Xoo* trains, the known resistance genes/QTLs to Asian *Xoo* strains which co-localized with QTL induced by African *Xoo* will be used in rice breeding programs to develop bacterial blight resistant cultivars for Africa.

## Conclusion

We mapped several resistance QTLs to bacterial blight in rice using a reference recombinant inbred lines derived from the cross between Azucena and IR64 rice varieties. Some of them are specific and novel to African or Asian strain of *Xanthomonas oryzae* pv. *oryzae* such as *qABB-9* induced by the African *Xoo* strain NAI8 on chromosome 9, *qBB-4* and *qBB-5* induced on chromosomes 4 and 5 by Asian *Xoo* strains PXO61 and PXO86, respectively. The others co-localize with known *Xoo* resistance genes/QTLs. It is the case of *qABB-1*, the resistance QTL induced by the African *Xoo* strain MAI1 on rice chromosome 1 which co-localize with *xa34(t)* resistance gene to Chinese *Xoo* races V. In the same way, *AQBT021* effective on Asian *Xoo* strains, co-localize with *qABB-10*, a resistance QTL induced by the African *Xoo* strain NAI8 on chromosome 10.

So far, and for the first time, specific resistance QTLs to African *Xoo* are identified and mapped. The fine mapping of the QTL induced by African *Xoo* MAI1 and BAI4 on chromosome 7 which controlled 37 % of phenotypic variance as well as the one induced on chromosome 11 by all African *Xoo* tested is ongoing and will provide news markers for breeding program.

## Methods

### Plant materials

The reference mapping population consisted in 172 recombinant inbred lines (RIL) obtained by single seed descent (SSD) from the cross between the tropical japonica landrace Azucena (susceptible parent) and the indica cultivar IR64 (resistant parent) was used to identify and map the bacterial blight (BB) resistance QTLs. IR64 is an elite improved indica rice grown in tropical Asia. It carries the BB resistance gene *Xa4* and has a large spectrum of resistance to *Xoo.* The Azucena landrace is susceptible to BB disease, and tolerant to drought and other abiotic stresses. The IR64 x Azucena mapping population was used to develop genetic map for characterizing gene/QTLs associated to drought, iron toxicity tolerance and resistance to the Rice yellow Mottle Virus (Boisnard A et al. 2007).

### Bacterial strains and plant inoculations

Six *Xanthomonas oryzae* pv. *oryzae* (*Xoo)* strains were used to evaluate resistance to BB in the mapping population. These strains included four African *Xoo* strains: BAI3 and NAI8 (race A1) from Burkina-Faso and Niger respectively, BAI4 (race A2) from Burkina-Faso, MAI1 (race A3) from Mali and two Asian strains PXO61 (Philippines race 1) and PXO86 (Philippines race 2). The bacterial strains were cultured on PSA medium (per liter of H_2_O, 10 g of peptone, 10 g of sucrose, 1 g of glutamic acid, and 16 g of bacto agar at pH 7.0) overnight. Inoculum was prepared by re-suspending bacterial culture in sterile, distilled water at an optical density of 0.2 (DO_600_), bearing approximately 10^8^ cfu per ml. The progenies and their parents were grown under controlled conditions (28 °C; 80 % humidity and 12 h day length) in greenhouse at IRD Montpellier/France. They were inoculated at the booting stage (6 weeks after germination) using the leaf-clipping method (Kauffman et al. 1973) in which the fully-opened leaves were cut with scissors dipped in the bacterial suspension. Ten plants per genotype and two uppermost fully expanded and randomly chosen leaves were inoculated with each strain. Phenotypic evaluations, consisting of the disease scoring, were done 3 weeks after inoculation by measuring the leaf lesion length. Disease reactions were classified according to the mean lesion length (LL) as following: resistant (R) if the lesion length was < 5 cm, moderately resistant (MR) with LL of 5 to 10 cm; moderately susceptible (MS) with LL of 10 to 15 cm, susceptible (S) with LL > 15 cm.

### Molecular mapping analysis using SSR markers

Leaves of rice plants were harvested and ground in liquid nitrogen. Genomic DNA was extracted as previously described (Edwards et al. 1991). PCR were performed in 15 μL reactions in an automated thermal cycler and the program consisted of the following cycles: initial denaturation at 94 °C for 5 min; 30 cycles of denaturation at 94 °C for 30 s, annealing temperature for 30 s and extension at 72 °C for 45 s; and a final extension step at 72 °C for 5 min. Amplified products were analyzed by electrophoresis on 6.5 % polyacrylamide gels, using electrophoresis system LICOR; or by electrophoresis in a 2 % agarose gel.

### Statistical analysis and *Xoo* resistance QTLs mapping

A linkage map comprising 226 SSR markers and constructed from the RIL population was used for mapping resistance QTL to *Xoo*. An analysis of variance, using marker genotypes as the groups, was carried out using MapDisto (Lorieux 2007). Data files were prepared using the Export map and data function of MapDisto. Analyses of distribution of the phenotypic traits as well as QTL detection were mainly performed using the Qgene v. 4.3.0 program (Nelson 2005, http://www.qgene.org) and Windows QTL cartographer 2.5 (Wang et al. 2006b). Different methods were compared such as Single-marker regression (SMR), Simple interval mapping (SIM), and Composite interval mapping (CIM). The Forward cofactor selection option was used in CIM. The LOD score statistic was used for all methods in order to make the results comparable. Empirical thresholds to declare presence of a QTL were obtained using the resampling by permutation method, performing 1,000 iterations for each trait/chromosome combination (log-likelihood of odds (LOD) score of 3).

### Heredity studies

#### QTL mapping using Asian *Xoo* strain PXO61

At the locus of *qABB-11*, the QTL on chromosome 11 that was involved in the resistance on all African *Xoo* tested, were localized a cluster of *Xa* genes including *Xa3, Xa4* and *Xa21. Xa3* was not effective against *Xoo* race 1 (Gonzalez et al. 2007). *Xa21* was identified in *Oryza longistaminata*, a wild rice race. Therefore, *Xa4* would be the only one *Xa* candidate gene at the above locus. In order to validate the presence of *Xa4* gene at this locus, the Asian *Xoo* strain PXO61 belonging to Philippines race 2 was used to screen the RIL population according to Kauffman et al. (1973). The resistance of rice to PXO61 strain was specifically under *Xa4* control.

#### Development and screening of F_2_: IR24 x IRBB4 population

The Asian rice variety IR24 belonging to indica sub-species was crossed with isogenic line IRBB4 carrying *Xa4* gene. IRBB4 was used as donor while IR24 represent a recurrent parent. The Hybrid F_1_ obtained was used to generate a F_2_ population. Two sets of 100 F_2_ individuals were screened with the African *Xoo* strain BAI3 and the Asian one PXO61, respectively.

#### In planta growth experiments

Rice variety IR24 with its isogenic line IRBB4 were screened using African *Xoo* strain BAI3 and Asian *Xoo* strain PXO61. Two, three and four pieces of 5 cm from the apex to the base of infected leaf were harvested 4, 8 and 12 days after inoculation, respectively. On each day, infected leaves fragments were harvested on three different plants. Infected leaves collected were briefly rinsed in 70 % of ethanol for 10 s followed by submersion in sterilized water. Leaves were put into 2 ml eppendorf tubes containing 2 metallic beads (ϕ = 3 mm), frozen by submersion into liquid nitrogen and ground into fine powder using the Qiagen Tissue Lyser system (30 rounds/s for 2 min). Ground material was resuspended in 1 ml of sterilized water and 10 μl drops of a dilution series were spotted onto PSA medium plate in triplicates. The plates were incubated at 28 °C until colonies could be counted. This experiment was performed three times.

### Mapping of known resistance gene/QTLs on the reference Nipponbare physical map

In a first step, information on all known BB resistance genes and QTLs was reviewed. This review included gramene accessions, number of genes/QTLs, their names, synonyms and symbols, the genetic populations in which they were mapped. Their donor’s parents as well as their genetic position and their co-localized markers in various mapping populations were also reported here. In the same way, physical positions were recorded if available (Supplementary data). The different genetic maps used were SSR Cornell 2000, RIL IR64 x Azucena, DH IR4 x Azucena, RIL Lemont x Tequin, RIL Zhenshan 97 x Minghui 63, JRGP RFLP 2000 Nipponbare x Kasalath, Cornell RFLP 2001 *O. sativa* x *O. longistaminata*, and the reference MSU7. Physical positions of the cloned genes such as *Xa1, Xa7, Xa10, Xa23, Xa26, Xa27, xa5, xa13* and *xa25* were directly reported on the integrative map. For the non cloned genes and QTLs, we chose the closest ones with known genetic positions among the co-localized maker and interpolated them on the reference Nipponbare MSU7 physical map. Other genes and QTLs with conflicting positions, as well as those that have been not mapped such as *Xa11, Xa12, Xa15, xa32(t)*, *AQBT026* and *AQBT030*, were not positioned on the integrative map.

## Abbreviations

ANOVA: analysis of variance
BB: bacterial blight
CFU: colony-forming units
CIM: composite interval mapping
DO: optical density
LL: lesion length
LOD: log-likelihood of odds
MR: moderately resistant
MS: moderately susceptible
PSA: peptone sucrose agar
QTL: quantitative trait loci
R: resistant
RIL: recombinant inbred lines
S: susceptible
SIM: simple interval mapping
SMR: single marker regression
SSD: single seed descent
SSR: simple sequence repeats
TAL: transcription activator-like
*Xoo*: *Xanthomonas oryzae* pv. *oryzae*

## References

[CR1] Basso A, Onasany A, Issaka S, Sido AY, Haougui A, Adam T, Séré Y, Saadou M (2011). Le flétrissement bactérien du riz au Niger: diversité pathologique d’isolats collectés sur les périmètres irrigués. J Appl Biosci.

[CR2] Bhasin H, Bhatia D, Raghuvanshi S, Lore J, Sahi G, Kaur B, Vikal Y, Singh K (2012). New PCR-based sequence-tagged site marker for bacterial blight resistance gene *Xa38* of rice. Mol Breed.

[CR3] Bimolata W, Kumar A, Sundaram RM, Laha GS, Qureshi IA, Reddy GA, Ghazi IA (2013). Analysis of nucleotide diversity among alleles of the major bacterial blight resistance gene *Xa27* in cultivars of rice (Oryza sativa) and its wild relatives. Planta.

[CR4] Blair MW, Garris AJ, Iyer AS, Chapman B, Kresovich S, McCouch SR (2003). High resolution genetic mapping and candidate gene identification at the *xa5* locus for bacterial blight resistance in rice (Oryza sativa L.). Theor Appl Genet.

[CR5] Boisnard A, Albar L, Thiéméle D, Tondeau M, Ghesquiere A (2007). Evaluation of genes from eIF4E and eIF4G multigenic families as potential candidates for partial resistance QTLs to Rice yellow mottle virus in rice. Theoretical and Applied Genetics.

[CR6] Century KS, Lagman RA, Adkisson M, Morlan J, Tobias R, Schwartz K, Smith A, Love J, Ronald PC, Whalen MC (1999). Developmental control of *Xa21*-mediated disease resistance in rice. Plant J.

[CR7] Cheema KK, Grewal NK, Vikal Y, Sharma R, Lore JS, Das A, Bhatia D, Mahajan R, Gupta V, Bharaj T, Singh K (2008). A novel bacterial blight resistance gene from *Oryza nivara* mapped to 38 kb region on chromosome 4 L and transferred to *Oryza sativa* L. Genet Res.

[CR8] Chen H, Wang S, Zhang Q (2002). A new gene for bacterial blight resistance in rice located on chromosome 12 identified from Minghui 63, and elite restorer line. Phytopathology.

[CR9] Chen S, Liu X, Zeng L, Ouyang D, Yang J, Zhu X (2011) Genetic analysis and molecular mapping of a novel recessive gene *xa34(t)* for resistance against Xanthomonas oryzae pv. oryzae. Theor Appl Genet. DOI 10.1007/s00122-011-1534-7 10.1007/s00122-011-1534-7 21274511

[CR10] Chu ZH, Fu BY, Yang H, Xu CG, Li ZK, Sanchez A, Park YJ, Bennetzen JL, Zhang QF, Wang SP (2006). Targeting *xa13*, a recessive gene for bacterial blight resistance in rice. Theor Appl Genet.

[CR11] Chu ZH, Yuan M, Yao LL, Ge XJ, Yuan B, Xu CG, Li XH, Fu BY, Li ZK, Bennetzen JL, Zhang QF, Wang SP (2006). Promoter mutations of an essential gene for pollen development result in disease resistance in rice. Genes Dev.

[CR12] Djedatin G, Ndjiondjop MN, Mathieu T, Vera Cruz CM, Sanni A, Ghesquière A, Verdier V (2011). Evaluation of African cultivated rice *Oryza glaberrima* for resistance to bacterial blight. Plant Dis.

[CR13] Edwards K, Johnstone C, Thompson C (1991). A simple and rapidmethod for the preparation of plant genomic DNA for PCR analysis. Nucleic Acids Res.

[CR14] Geffroy V, Sevignac M, d’Oliveira J, Fouilloux G, Skroch P, Thoquet P, Gepts P, Langin T, Dron M (2000). Inheritance of partial resistance against *Colletotrichum lindemuthianum* in *Phaseolus vulgaris* and co-localization of quantitative trait loci with genes involved in specific resistance. Mol Plant Microbe Interact.

[CR15] Ghazi IA, Srivastava PS, Dalal V, Gaikwad K, Singh AK, Sharma TR, Singh NK, Mohapatra T (2009). Physical mapping, expression analysis and polymorphism survey of resistance gene analogues on chromosome 11 of rice. J Biosci.

[CR16] Gonzalez C, Szurek B, Manceau C, Mathieu T, Sere Y, Verdier V (2007). Molecular and pathotypic characterization of new Xanthomonas oryzae strains from West Africa. Mol Plant Microbe Interact.

[CR17] Gu K, Tian D, Yang F, Wu L, Sreekala C, Wang D, Wang GL, Yin Z (2004). High-resolution genetic mapping of *Xa27(t)*, a new bacterial blight resistance gene in rice, Oryza sativa L. Theor Appl Genet.

[CR18] Hajri A, Brin C, Zhao S, David P, Feng J, Koebnik R, Szurek B, Verdier V, Boureau T, Poussier S (2012) Multilocus sequence analysis and type III effector repertoire mining provide new insights into the evolutionary history and virulence of Xanthomonas oryzae. Molecular Plant Pathology 13:288–302 10.1111/j.1364-3703.2011.00745.x PMC6638859 21929565

[CR19] Han X, Yang Y, Wang X, Zhou J, Zhang W, Yu C, Cheng C, Cheng Y, Yan C, Chen J (2014). Quantitative Trait Loci Mapping for Bacterial Blight Resistance in Rice Using Bulked Segregant Analysis. Int J Mol Sci.

[CR20] Iyer AS, McCouch SR (2004). The rice bacterial blight resistance gene *xa5* encodes a novel form of disease resistance. Mol Plant Microbe Interact.

[CR21] Kauffman HE, Reddy APK, Hsieh SPY, Merca SD (1973). An improved technique for evaluating resistance to rice varieties of Xanthomonas oryzae. Plant Dis Rep.

[CR22] Kawahara Y (2013). Improvement of the Oryza sativa Nipponbare reference genome using next generation sequence and optical map data. Rice.

[CR23] Kim S, Suh J, Qin Y, Noh T, Reinke RF, Jena KK (2015) Identification and fine-mapping of a new resistance gene, Xa40, conferring resistance to bacterial blight races in rice (Oryza sativa L.) Theoretical and Applied Genetics 128:1933–1943. 10.1007/s00122-015-2557-2 26081948

[CR24] Korinsak S, Sriprakhon S, Sirithanya P, Jairin J, Korinsak S, Vanavichit A, Toojinda T (2009). Maejo Int J Sci Technol.

[CR25] Lee SW, Choi SH, Han SS, Lee DG, Lee BY (1999). Distribution of Xanthomonas oryzae pv. oryzae strains virulent to *Xa21* in Korea. Phytopathology.

[CR26] Li ZK, Luo LJ, Mei HW, Paterson AH, Zhao XZ, Zhong DB, Wang YP, Yu XQ, Zhu L, Tabien R, Stansel JW, Ying CS (1999). A “defeated” rice resistance gene acts as a QTL against a virulent strain of *Xanthomonas oryzae* pv. *oryzae*. Mol Gen Genet.

[CR27] Liu Q, Yuan M, Zhou Y, Li X, Xiao J, Wang S (2011). A paralog of the MtN3/saliva family recessively confers race-specific resistance to *Xanthomonas oryzae* in rice. Plant Cell Environ.

[CR28] Lorieux M (2007). MapDisto, A Free User-Friendly Program For Computing Genetic Maps. Computer demonstration (P958) given at the Plant and Animal Genome XV conference, Jan 13-17 2007, San Diego, CA.

[CR29] Mago R, Nair S, Mohan M (1999). Resistance gene analogues from rice: cloning, sequencing and mapping. Theor Apply Genet.

[CR30] Mew TW, Vera Cruz CM, Medalla ES (1992). Changes in race frequency of Xanthomonas oryzae pv. oryzae in response to the planting of rice cultivars in the Philippines. Plant Dis.

[CR31] Nelson J (2005) Methods and Software for Genetic Mapping, pp. 53–74 in The Handbook of Plant Genome Mapping; Genetic and Physical Mapping, edited by Meksem K., Kahl G., editors. Wiley-VCH Verlag GmbH and Co, Weinheim, Germany

[CR32] Ogawa T, Yamamoto T (1987). Selection of Recurrent Parents to Develop near-Isogenic Lines Resistant to Bacterial Leaf-Blight of Rice. Jarq Japan Agric Res Q.

[CR33] Perez LM, Redoña ED, Mendioro MS, Vera Cruz CM, Leung H (2008). Introgression of Xa4, Xa7 and Xa21 for resistance to bacterial blight in thermosensitive genetic male sterile rice (Oryza sativa L.) for the development of two-line hybrids. Euphytica.

[CR34] Pflieger S, Lefebvre V, Caranta C, Blattes A, Goffinet B, Palloix A (1999). Disease resistance gene analogs as candidates for QTLs involved in pepper–pathogen interactions. Genome.

[CR35] Ramalingam J, Cruz CMV, Kukreja K, Chittoor JM, Wu JL, Lee SW, Baraoidan M, George ML, Cohen MB, Hulbert SH, Leach JE, Leung H (2003). Candidate Defense genes from rice, barley, and maize and their association with qualitative and quantitative resistance in rice. Mol Plant Microbe Interact.

[CR36] Reckhaus PM (1983). Occurrence of bacterial blight of rice in Niger, West Africa. Plant Dis.

[CR37] Ruan H,Yan C, An D, Liu R, Chen J (2008) Identifying and Mapping New Gene *xa32(t)* for Resistance to Bacterial Blight(*Xanthomonas oryzae* pv. *oryzae, Xoo*) from Oryza meyeriana L. Acta Agriculturae Boreali-Occidentalis Sinica. DOI CNKI:SUN:XBNX.0.2008-06-035

[CR38] Ruengphayak S, Chaichumpoo E, Phromphan S, Kamolsukyunyong W, Sukhaket W, Phuvanartnarubal E, Korinsak S, Korinsak S, Vanavichit A (2015). Pseudo-backcrossing design for rapidly pyramiding multiple traits into a preferential rice variety. Rice.

[CR39] Sakraborty S, Zeng Z (2011). QTL Mapping for Days to Flowering under Drought Condition in Rice (Oryza sativa L.) Genome. Notulae Botanicae Horti Agrobotanici Cluj-Napoca.

[CR40] Sanchez AC, Ilag LL, Yang D, Brar DS, Ausubel F, Khush GS, Yano M, Saskai T, Li Z, Huang N (1999). Genetic and physical mapping of *xa13*, a recessive bacterial blight resistance gene in rice. Theor Appl Genet.

[CR41] Sere Y, Onasanya A, Verdier V, Akator K, Ouedraogo LS, Segda Z, Mbare MM, Sido AY, Baso A (2005). Rice Bacterial Leaf Blight in West Africa: Preliminary studies on disease in farmer’s field and screening. Asian J Plant Sci.

[CR42] Shanti ML, Shenoy VV, Lalitha Devi G, Mohan Kumar V, Premalatha P, Naveen Kumar G, Shashidhar HE, Zehr UB, Freeman WH (2010). Marker-assisted breeding for resistance to bacterial leaf blight in popular cultivar and parental lines of hybrid rice. J Plant Pathol.

[CR43] Singh S, Sidhu JS, Huang N, Vikal Y, Li Z, Brar DS, Dhaliwal HS, Khush GS (2006). Pyramiding three bacterial blight resistance genes (xa5, xa13 and Xa21) using marker-assisted selection into indica rice cultivar PR106. Theoretical and Applied Genetics..

[CR44] Suh JP, Jeung JU, Noh TH, Cho YC, Park SH, Park HS, Shin MS, Kim CK, Jena KK (2013). Development of breeding lines with three pyramided resistance genes that confer broad-spectrum bacterial blight resistance and their molecular analysis in rice. Rice.

[CR45] Sun XL, Cao YL, Yang ZF, Xu CG, Li XH, Wang SP, Zhang QF (2004). *Xa26*, a gene conferring resistance to *Xanthomonas oryzae* pv. *oryzae* in rice, encodes an LRR receptor kinase-like protein. Plant J.

[CR46] Sundaram RM, Vishnupriya MR, Laha GS, Rani NS, Rao PS, Balachandran SM, Reddy GA, Sarma NP, Sonti RV (2009). Introduction of bacterial blight resistance into Triguna, a high yielding, mid-early duration rice variety. Biotechnol J.

[CR47] Swamy P, Panchbhai AN, Dodiya P, Naik V, Panchbhai SD, Zehr UB, Azhakanandam K, Char BR (2006). Evaluation of bacterial blight resistance in rice lines carrying multiple resistance genes and *Xa21* transgenic lines. Curr Sci.

[CR48] Tian D, Wang J, Zeng X, Gu K, Qiu C, Yang X, Zhou Z, Goh M, Luo Y, Murata-Hori M (2014). The rice TAL effector-dependent resistance protein *XA10* triggers cell death and calcium depletion in the endoplasmic reticulum. Plant Cell.

[CR49] Toojinda T, Broers LH, Chen XM, Hayes PM, Kleinhofs A, Korte J, Kudrna D, Leung H, Line R, Powell W, Ramsey L, Vivar H, Waugh R (2001). Mapping quantitative and qualitative disease resistance genes in a doubled haploid population of barley (*Hordeum vulgare*). Theor Appl Genet.

[CR50] Traoré O, Pinel-Galzi A, Sorho F, Sarra S, Rakotomalala M, Sangu E, Kanyeka Z, Séré Y, Konaté G, Fargette D (2009). A reassessment of the epidemiology of Rice yellow mottle virus following recent advances in field and molecular studies. Virus Res.

[CR51] Triplett LR, Hamilton JP, Buell CR, Tisserat NA, Verdier V, Zink F, Leach JE (2011). Genomic Analysis of *Xanthomonas oryzae* from US Rice Reveals Substantial Divergence from Known X. oryzae Pathovars. Appl Environ Microbiol.

[CR52] Vera Cruz CM, Bai J, Oña I, Leung H, Nelson RJ, Mew T, Leach JE (2000). Predicting durability of a disease resistance gene based on an assessment of the fitness loss and epidemiological consequences of avirulence gene mutation. PNAS.

[CR53] Verdier V, Vera Cruz C, Leach JE (2012) Controlling rice bacterial blight in Africa: Needs and prospects. J Biotechnol 159(4):320-328 10.1016/j.jbiotec.2011.09.020 21963588

[CR54] Wang G, Mackill DJ, Bonman JM, McCouch SR, Nelson RJ (1994). RFLP mapping of genes conferring complete and partial resistance to blast resistance in a durably resistant rice cultivar. Genetics.

[CR55] Wang G, Wan X, Crossa J, Crouch J, Weng J, Zhai H, Wan J (2006). QTL mapping of grain length in rice (Oryza sativa L.) using chromosome segment substitution lines. Genet Res.

[CR56] Wang S, Basten CJ, Zeng ZB (2006). Windows QTL Cartographer2.5.

[CR57] Wang CL, Xu AB, Gao Y, Fan YL, Liang YT, Zheng CK, Sun LQ, Wang WQ, Zhao KJ (2009). Generation and characterisation of Tn5-*tagged Xanthomonas oryzae* pv. *oryzae* mutants that overcome *Xa23-*mediated resistance to bacterial blight of rice. Eur J Plant Pathol.

[CR58] Wang C, Fan Y, Zheng C, Qin T, Zhang X, Zhao K (2014). High-resolution genetic mapping of rice bacterial blight resistance gene Xa23. Mol Genet Genomics.

[CR59] Wang M, Yu Y (2014). The genome sequence of African rice (Oryza glaberrima) and evidence for independent domestication. Nat Genet.

[CR60] Webb KM (2010). A benefit of high temperature: Increased effectiveness of a rice bacterial blight disease resistance gene. New Phytol.

[CR61] Wonni I, Cottyn B, Detemmerman L, Dao S, Ouedraogo L, Sarra S, Tekete C, Poussier S, Corral R, Triplett L, Koita O, Koebnik R, Leach J, Szurek B, Maes M, Verdier V (2014). Analysis of *Xanthomonas oryzae* pv. *oryzicola* population in Mali and Burkina Faso reveals a high level of genetic and pathogenic diversity. Phytopathology.

[CR62] Wu L, Goh ML, Sreekala C, Yin Z (2008). *XA27* Depends on an Amino-Terminal Signal-Anchor-Like Sequence to Localize to the Apoplast for Resistance to *Xanthomonas oryzae* pv *oryzae*. Plant Physiol.

[CR63] Wu X, Li X, Xu C, Wang S (2008). Fine genetic mapping of *xa24*, a recessive gene for resistance against *Xanthomonas oryzae* pv. *oryzae* in rice. Theor Appl Genet.

[CR64] Xiang Y, Cao YL, Xu CG, Li XH, Wang SP (2006). *Xa3*, conferring resistance for rice bacterial blight and encoding a receptor kinase-like protein, is the same as *Xa26*. Theor Appl Genet.

[CR65] Yoshimura S, Yamanouchi U, Katayose Y, Toki S, Wang ZX, Kono I, Kurata N, Yano M, Iwata N, Sasaki T (1998). Expression of *Xa1*, a bacterial blight-resistance gene in rice, is induced by bacterial inoculation. Proc Natl Acad Sci U S A.

[CR66] Yu Y, Streubel J, Balzergue S, Champion A, Boch J, Koebnik R, Feng J, Verdier V, Szurek B (2011). Colonization of rice leaf blades by an African strain of *Xanthomonas oryzae* pv. *oryzae* depends on a new TAL effector which induces the rice nodulin-3 *Os11N3* gene. Mol Plant Microbe Interact.

[CR67] Yu Y, Streubel J, Balzergue S, Champion A, Boch J, Koebnik R, Feng J, Verdier V, Szurek B (2011) Colonization of rice leaf blades by an African strain of *Xanthomonas oryzae* pv. *oryzae* depends on a new TAL effector which induces the rice nodulin-3 *Os11N3* gene. Mol Plant Microbe Interact. Epub ahead of print 10.1094/MPMI-11-10-0254 21679014

[CR68] Zhang J, Li X, Jiang G, Xu Y, He Y (2006). Pyramiding of *Xa7* and *Xa21* for the improvement of disease resistance to bacterial blight in hybrid rice. Plant Breed.

[CR69] Zou JH, Pan XB, Chen Z, Xu JY, Lu JF, Zhai WX, Zhu LH (2000). Mapping quantitativre trait loci controlling sheath blight resistance in two rice cultivars (Oryza sativa L.). Theor Appl Genet.

